# Selective activity of *Tabebuia avellanedae* against *Giardia duodenalis* infecting organoid-derived human gastrointestinal epithelia

**DOI:** 10.1016/j.ijpddr.2025.100583

**Published:** 2025-01-22

**Authors:** Giulia Rigamonti, Fabrizia Veronesi, Elisabetta Chiaradia, Petra Gosten-Heinrich, Antonia Müller, Leonardo Brustenga, Stefano de Angelis, Alessia Tognoloni, Riccardo De Santo, Christian Klotz, Marco Lalle

**Affiliations:** a Department of Veterinary Medicine, University of Perugia, via San Costanzo 4, Perugia, Italy; b Department of Infectious Diseases, Unit 16 Mycotic and Parasitic Agents and Mycobacteria, Robert Koch-Institute, Seestrasse 20, Berlin, Germany; c Deakos SRL, Corso Nazionale 169, La Spezia, Italy; d Department of Chemistry, Sapienza University, P.le Aldo Moro, 5. 00185, Rome, Italy; e Department of Infectious Diseases, Unit of Foodborne and Neglected Parasitic Diseases, Istituto Superiore di Sanità, viale Regina Elena 299, Rome, Italy

**Keywords:** *Giardia duodenalis*, *Tabebuia avellanedae*, *In vitro* activity, Safety, Intestinal organoid-derived monolayer (ODM)

## Abstract

*Giardia duodenalis* is a widespread intestinal protozoan that affects mammals, including humans. Symptoms can range from being subclinical to causing severe abdominal pain and diarrhoea. Giardiasis often requires repeated treatment with synthetic drugs like metronidazole. In recent years, treatment failures in clinical cases involving nitroimidazoles have been increasingly reported. Consequently, identifying therapeutic alternatives is necessary. Medicinal plants have traditionally been used as antiparasitic compounds, but systematic evaluation under controlled experimental conditions is often lacking. Here, we evaluated the *in vitro* efficacy of *Tabebuia avellanedae* dry and hydroalcoholic extracts, as well as one of its active compounds, β-lapachone, as potential treatment against *G. duodenalis* infection. We observed effective antigiardial activity for all tested compounds, with β-lapachone exhibiting lower IC_50_ values than metronidazole. Cytotoxic effects often limit therapeutic concentration windows of opportunity, and choosing an informative model to assess them is not straightforward. In the present case, only *T. avellanedae* hydroalcoholic extract showed no cytotoxicity on tumoral human intestinal Caco-2 cell line, and only a trend of inhibition when tested on canine epithelial kidney MDCK cells. To introduce a more physiological test system, we used *in vitro G. duodenalis* infection experiments in a trans-well set-up using organoid derived monolayers (ODM) to assess at the same time drug efficacy against the parasite and safety on primary human intestinal epithelia, a likely surrogate for *in vivo* conditions. Our studies using this model point towards the potential therapeutic opportunity for non-systemic applications of *T. avellanedae* extracts and a relevant ingredient of these, β-lapachone. The data suggest that ODM co-cultures with *G. duodenalis* are suitable for testing antigiardial compounds, providing a more informative *in vitro* model before progressing to *in vivo* tests.

## Highlights

• Giardiasis is related to treatment failures, and no human vaccine is available.
• There is a need for new therapeutic allies.
• *Tabebuia avellanedae* and β-lapachone are effective against *Giardia duodenalis.*
• Organoid-derived monolayers are a promising model for testing antigiardial drugs.

## Graphical abstract

**Figure:**
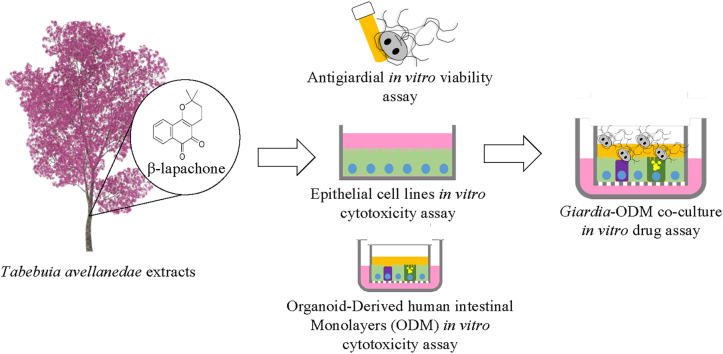

## 1 Introduction

*Giardia duodenalis* (syn. *G. intestinalis* or *G. lamblia*) is a widespread protozoan parasite able to infect the proximal small intestine of more than 40 mammals, including humans (Lalle and Hanevik, 2018). Infection occurs via the faecal-oral route through the accidental ingestion of *G. duodenalis* cysts, the environmentally resistant stage of the parasite, either by direct contact with infected faeces or by ingestion of contaminated water or food (such as fresh produce). The waterborne route is most often associated with human infection. *G. duodenalis* can be classified in eight groups, or Assemblages, based on the genetic profiles and host specificity. Assemblages A and B display a certain level of zoonotic potential, as they can be isolated from both humans and animals (especially sub-assemblage AI and B), whereas Assemblages C and D are only found in canids, E in ungulates, F in felids, G in rodents and H in pinnipeds (Cacciò et al., 2018). In humans, infection can reach prevalence rates of 2–7% in high income countries and 20–60% in low-income countries and is generally high in settings with poor hygiene (Minetti et al., 2016; Fantinatti et al., 2021). Infection can range from asymptomatic cases to chronic diarrhoea, malabsorption, and weight loss (Ankarklev et al., 2010). Moreover, *G. duodenalis* interferes with the body's ability to absorb fat, lactose, vitamin A, and vitamin B12, causing progressive weight loss, malnutrition conditions and cognitive development problems, especially in children (Berkman et al., 2002; Allain et al., 2017). In humans no effective vaccine is available; approved drugs include six classes of compounds: 5-nitroimidazoles, benzimidazoles derivatives, quinacrine, furazolidone, paromomycin and nitazoxanide (Lalle and Hanevik, 2018). The 5-nitroimidazole metronidazole (MTZ) is the first-choice treatment, with recovery rates exceeding 90% (Gardner and Hill, 2001; Leung et al., 2019). Despite its efficacy, this molecule may exhibit adverse side effects (e.g., headache, nausea, glossitis, neutropenia) and has carcinogenic, teratogenic, and embryogenic properties (Gardner and Hill, 2001; Hernández Ceruelos et al., 2019). In recent years, treatment failures with MTZ have been reported in 10–20% of patients (Ansell et al., 2015; Argüello-García et al., 2020), and an increasing number of cases of giardiasis refractory to treatment, mainly with 5-nitroimidazoles, have been observed in humans. This suggests that mechanisms of tolerance to all major antigiardial drugs may not be uncommon in the parasite (Upcroft and Upcroft, 2001; Leitsch, 2015; Argüello-García et al., 2020). Therefore, new therapeutic alternatives for giardiasis are currently being investigated. For instance, new potential compounds are being identified based on ethnopharmacological concepts regarding the efficacy of medicinal plants and/or their active molecules or structurally similar analogues (Cragg and Newman, 2005; Wink, 2012; de Almeida et al., 2020). *Tabebuia avellanedae* (Lorentz ex Griseb.) (syn. *Tabebuia impetiginosa* or *Handroanthus impetiginosus* or *Red Lapacho*) (Fam. Bignoniaceae) is a tree native to Central and South America, widely used in local and traditional phytomedicine to treat bacterial, fungal, and protozoan infections (Zhang et al., 2020). Traditionally, *T. avellanedae* is consumed as tea, made from the inner bark of the tree. The traditional preparation involves making a decoction from half to one cup of bark and/or wood, taken orally two to four times a day (Taylor, 2005). Further, *T. avellanedae* applications include douching to treat yeast infections; topical application to treat fungal skin infections; use of tincture, ingested orally or applied to mucous membranes to reduce inflammation (Gómez Castellanos et al., 2009). In addition, the main bioactive components of *T. avellanedae*, naphthoquinones (e.g., lapachol, β-lapachone, and α-lapachone), have shown cytotoxicity against several tumor cell lines *in vitro* (Boothman et al., 1988; Huang and Pardee, 1999; Li et al., 2023) and *in vivo* (Kim et al., 2017; Gerber et al., 2018), as well as antibacterial activity (e.g., against *Clostridium perfringens, Escherichia coli,* and *Helicobacter pylori*) (Park et al., 2005, 2006) and anti-protozoan activity against *Trypanosoma cruzi*, *Leishmania* spp., and *Plasmodium falciparum,* in both *in vivo* and *in vitro* studies (Pinto et al., 2000; Menna-Barreto et al., 2005; Araújo et al., 2019; do Nascimento et al., 2020; Mohammadi et al., 2020). Noteworthy, previous research showed that β-lapachone induces *in vitro* cell death and morphological abnormalities in *G. duodenalis* and stimulate the expression of encystation markers (Corrêa et al., 2009).

Here, we evaluated the antigiardial efficacy of *T. avellanedae* bark extracts and its bioactive compound β-lapachone. We also tested their cytotoxicity on common cell lines and characterized the metabolite profile of *T. avellanedae* extracts. Furthermore, the effect of these compounds was compared under more physiological conditions using a well-established stem cell-derived intestinal organoid model on trans-well filter as a surrogate infection model.

## 2 Materials and methods

### 2.1 Natural and chemical compounds

*Tabebuia avellanedae* dry extract (TD) and hydroalcoholic extract (EtOH 45%) (TH) were provided by Deakos SRL (La Spezia, IT). β-lapachone (2,2-Dimethyl-3,4-dihydro-2H-benzo [h]chromene-5,6-dione, C_15_H_14_O_3_) (L2037) and metronidazole (MTZ) (M3761) were purchased from Merck (Sigma-Aldrich, Milan, IT). For stock solutions, β-lapachone, MTZ and TD were dissolved in dimethyl sulphoxide (DMSO) at a concentration of 10 mM for β-lapachone and MTZ, and 200 mg/ml for TD; *Tabebuia avellanedae* hydroalcoholic extract was stocked undiluted at 100 mg/ml, according to the manufacturer's datasheet. All compounds were stored in dark and frozen condition, except for TH, which was stored at room temperature according to the manufacturer's instructions.

### 2.2 Parasite isolates and cultivation

The *G. duodenalis* isolates used in this study are listed in Table 1. Trophozoites were axenically grown at 37 °C in flat-sided 10 mL screw-cap tubes (Nunclon; Thermo Fischer Scientific, Waltham, MA, USA) filled with at least 10 mL filter-sterilized Keister's modified TYI-S-33 medium, supplemented with 10% adult bovine serum (Gibco, Grand Island, NY), 100 μg/mL streptomycin and 100 U/mL penicillin (Capricorn, Frederick, MD) and 0.05% bovine bile (Sigma-Aldrich, St. Louis, MO) (Keister, 1983). Confluent cultures were sub-cultured two to three times a week by placing tubes on ice for 20 min to detach trophozoites, then transferring 1:10–1:100 of the culture to fresh medium.

**Table 1:** List of *Giardia duodenalis* isolates used in the present work.

Isolate	Assemblage	Laboratory^a^	Reference
WBC6 (ATCC-50803)	AI	IT, DE	
GS/M (ATCC-50581)	B	IT, DE	
P424/A5	B	DE	Klotz et al. (2023)

a = IT=Italy, DE = Germany.

### 2.3 Mammalian cells lines and culture

Human colon adenocarcinoma cell lines (Caco-2) were cultured in Dulbecco's Modified Eagle Medium (DMEM) supplemented with 10% inactivated foetal bovine serum (FBS), 1% L-glutamine, 1% penicillin-streptomycin and 1% non-essential amino acids solution (Natoli et al., 2012; Awortwe et al., 2014). Madin-Darby canine kidney cells (MDCK) were cultured in the same medium conditions but without the addition of non-essential amino acids solution (Rodrigues et al., 2020). Cells were cultivated at 37 °C in a humidified 5% CO_2_ atmosphere until reaching approximately 80% confluence before passage. Cell culture media and supplements were all purchased from Euroclone (Milan, IT).

### 2.4 Intestinal organoid derived monolayers (ODM)

Human duodenal Organoid-Derived Monolayers (ODM) were prepared from an established and well-characterized organoid culture, as previously described (Holthaus et al., 2021, 2022; Warschkau et al., 2022; Weiss et al., 2022). Briefly, ODM were generated on Cultrex-coated (Bio-Techne, Minneapolis, MN, USA) trans-well cell culture inserts (0.6 cm^2^, 0.4 μm pores; Merck-Millipore, Burlington, MA, USA). To achieve this, 3D stem cell-enriched intestinal organoid cultures (previously obtained from small intestine biopsy specimen from a healthy volunteer at Charité Universitätsmedizin Berlin, with ethics approval #EA4-015-13 by German authorities, for details see Holthaus et al., 2022) were collected in Advanced DMEM/F-12, centrifuged, and mechanically disrupted to generate a single-cell suspension, following exactly the previously established procedure (Warschkau et al., 2022). The resulting cells were added to the apical compartment of the insert (5x10^5^ cells/filter). To obtain ODM with a physiological cell composition, consisting mainly of enterocytes (Holthaus et al., 2022), differentiation was induced using ODM differentiation medium. This medium consisted of 20% (v/v) R-Spondin 1-conditioned medium, 10% (v/v) Noggin-conditioned medium, 50 ng/mL Human Epidermal Growth Factor (hEGF), 1 mM HEPES, 2 mM GlutaMax, 1 × P/S (100 U/mL penicillin and 100 μg/mL streptomycin), 1 × N2, 1 × B27, 1 mM N-acetyl-L-cysteine, and 10 mM nicotinamide in Advanced DMEM/F-12 (Holthaus et al., 2022; Warschkau et al., 2022). Both compartments of the trans-well system received the ODM differentiation medium, which was refreshed every 2–3 days (Holthaus et al., 2022). Transepithelial electric resistance (TEER) measurements were made on a 37 °C heating block using a Millicell ERS-2 Voltohmmeter (Merck-Millipore) that was equipped with an Ag/AgCl electrode (STX01; Merck-Millipore) (Holthaus et al., 2022; Warschkau et al., 2022). After 8 days, the ODM reached a confluent stage, as confirmed by TEER, and were used for subsequent applications.

### 2.5 In vitro cytotoxicity assays

The drug susceptibility of *G. duodenalis* trophozoites was determined using a previously described bioluminescent ATP content assay (Chen et al., 2011) with some modifications. The assays were carried out in 96-well microplates with flat clear bottoms. Trophozoites from a log-phase culture were harvested by chilling on ice and counted in a hemocytometer (Kova™, Thermo Fischer Scientific, Waltham, MA, USA). Trophozoites were seeded at 0.5x10^5^/well in 200 μl of modified TYI-S-33 medium. Two-fold serial dilutions of the compounds were prepared in DMSO or EtOH 45% at 100X concentration and individually added to each well. All wells contained DMSO 1% and EtOH 0.45%. Cells were incubated at 37 °C for 48 h under anaerobic conditions by placing the microplates in Aerogen Oxoid jars containing appropriate reaction bags (Oxoid #AN0025). After 48 h, trophozoites viability was determined by the bioluminescent ATP content assay, according to the manufacturer's instructions (CellTiterGlo 2.0, Promega Italia, Milan, IT). Each experiment was done in triplicate, and at least three biological replicates were performed.

To evaluate drug cytotoxicity against mammalian cells, Caco-2 and MDCK cells were seeded onto 96-well microplates in 100 μL of culture medium at a density of 1x10^4^ cell/well and 1.5x10^4^ cell/well, respectively. Both cell lines were allowed to attach to the plate for 24 h. After 24 h, the medium was replaced with fresh medium containing the same compounds at the same concentrations used for testing *G. duodenalis* trophozoites. Each concentration was tested in triplicate, and viability measured at 6, 12, 24 or 48 h. At the end of incubation time, 10 μL/well of Cell Counting Kit-8 (CCK8) solution (Target Molecule Corp, Boston, USA) was added and incubated at 37 °C for 2 h for Caco-2 and 1 h for MDCK until the colour turned orange. CCK-8 is an assay for assessing cell viability and cytotoxicity; it utilises WST-8 [2-(2-methoxy-4-nitrophenyl)-3-(4-nitrophenyl)-5-(2,4-disulfophenyl)-2H-tetrazolium, monosodium salt], a water-soluble tetrazolium salt reduced by cellular dehydrogenases to a coloured formazan dye. The amount of formazan generated is directly proportional to the number of living cells. The absorbance was measured using a plate spectrophotometer (Infinite 200 Pro-Tecan, Mennedorf, CH) at 450 nm. All 96-well microplates included media-only, and vehicle (DMSO and EtOH) control. Each experiment was done in triplicate, and at least three biological replicates were performed.

### 2.6 UHPLC-IMS-HRMS analysis of TH

To investigate the metabolite profile of TH, an untargeted screening was performed. For this purpose, a diluted aliquot of TH (1:100) in a 70% ethanol solution was analysed on an ACQUITY UHPLC I-Class system (Waters Corporation) coupled to a Synapt XS HDMS mass spectrometer (Waters Corporation) using an electrospray ionization interface operating in positive mode (ESI+). Data acquisition and processing were performed using UNIFI software (Version 3.1.0.16, Waters Corporation). Chromatography separation was performed using a BEH C18 column (2.1 × 100 mm, 1.7 μm particle size; Waters Corporation), mobile phases A = Water (LC-MS grade purchased from Sigma-Aldrich) and B = Acetonitrile (LC-MS grade purchased from Sigma-Aldrich), both containing 0.1% of formic acid (purchased from Sigma-Aldrich). The gradient started with 20% acetonitrile and reached 100% in 10 min; then, the system returned to initial conditions in 1 min and finally equilibrated for 2 min, resulting in a total runtime of 13 min. A flow rate of 0.4 mL/min, a column oven temperature of 40 °C, and a sample injection volume of 2 μL was selected. A vial containing 100 μL of the 70% ethanol solution was analysed under the same conditions as the TH aliquot and was used as a procedural blank to remove solvents and system interferences.

After chromatographic separation, the metabolites were detected by the combination of ion mobility separation coupled to high-resolution mass spectrometry (IMS-HRSM). Regarding the IMS-HRMS setup, the capillary voltage was set at 2 kV, the source temperature was set to 120 °C, and the desolvation gas temperature was set to 20 °C with a flow rate of 300 L/h. The mass spectrometer was operated in ion mobility mode (HDMS^E^) for acquisition. HDMS^E^ experiments provide the acquisition of a low-energy function (LE) and high energy function (HE); 6 eV was set as collision energy for the LE function, and the collision energy ramp from 25 to 50 was set for the HE function. HDMS^E^ is a data-independent acquisition method that can determine the drift time (DT) of each ion. DT can be converted into a collision cross-section value (CCS, Å^2^), which provides information about the structure of a chemical compound and its three-dimensional conformation (Mairinger et al., 2018). The CCS value represents an additional level of identification for unknown compounds. The combination of ion mobility experiments with accurate mass determination and mass spectra allows for the elucidation of unknown substances in real samples with a high level of confidence. Calibrations of mass axis and CCS were performed monthly using a “Major Mix Calibration Sample Kit” supplied by the vendor (Waters Corporation). A Leucine-Enkephalin solution (100 ppb, purchased from Waters Corporation) in ACN:H_2_O (50:50, *v/v*) with 0.01% of formic acid was used as lock mass to ensure the robust accurate mass measurement throughout runs.

The Synapt instrument data was imported to UNIFI software. The following key parameters were set on the UNIFI platform for data processing: mass error 10 ppm, fragment match tolerance 2 mDa, look for in-source fragments, maximum candidates per sample to keep: 10,000, adducts to search: H^+^. The “Binary Sample Compare” tool was applied on UNIFI between the TH and procedural blank spectra. This approach allowed for the identification of unique metabolites present in TH by subtracting the compounds present in the procedural blank from those present in TH. For the elucidation of TH metabolites, the experimental results were compared with the theoretical information available in the “Natural Products Profiling CCS Library”, an online library integrated into UNIFI software that contains compounds belonging to the following compound classes: alkaloids, coumarins, flavones, isoflavones, macrolides, peptides, synthetic derivatives, and terpenoids. Only substances whose m/z and CCS values, as well as mass spectra, molecular formulas, and structures matched those contained in the library were selected as possible metabolites.

### 2.7 Cytotoxicity evaluation on non-infected ODM and ODM infected with G. duodenalis trophozoites

For cytotoxicity assays, the compounds were added to the apical compartment medium of 8-day-old ODM at final concentration of 2, 1, and 0.25 mg/ml for TD; 1, 0.5, and 0.25 mg/ml for TH; and 100, 25, and 2.5 μM for β-lapachone. As a positive control for cell death, 2 μM staurosporine (Tocris, Bristol, UK), a non-selective apoptosis inducer, was used in all experiments (Holthaus et al., 2022). After 48 h, cell viability was evaluated using a luminescent assay that assesses the quantity of ATP present in the culture as an indicator of metabolically active cells (CellTiterGlo 2.0, Promega, DE). Furthermore, to test the effects of TH and β-lapachone on ODM infected with *G. duodenalis*, the apical compartment medium of 8-day-old ODM was replaced with TYI-S-33 the evening before infection to adapt the cells to the *G. duodenalis* medium. TYI-S-33 was renewed prior to infection (i.e., TYI-S-33 in the apical compartment and organoid differentiation medium in the basal compartment), and 2x10^5^ trophozoites (WBC6 strain) were added apically to obtain an approximate multiplicity of infection (MOI) of 1 (Holthaus et al., 2022). After that, the compounds were added to the apical compartment medium at concentrations of 1, 0.5, and 0.25 mg/ml for TH, and 100, 25, and 2.5 μM for β-lapachone. After 48 h, trophozoites were harvested by incubating the plates on ice for at least 20 min, followed by counting using a Neubauer cell counting chamber (Kova™, Thermo Fisher Scientific, Waltham, MA, USA). For both non-infected and infected ODM experiments, TEER measurements were made on the day of the treatment (day 0) and 48 h after the treatment (day 2). At the end of the experiment, selected filters were fixed with 4% paraformaldehyde for 15 min, washed in PBS, and used for immunofluorescence analysis (IFA) to confirm the integrity of the cell monolayer. For IFA, cells were permeabilized with 0.025% TritonX-100 in PBS for 30 min, followed by blocking with 1% BSA in PBS for 1 h and subsequent staining using anti-ZO1 antibody (1:250 dilution, #610967, BD Biosciences) in blocking buffer overnight at 4 °C. For detection and counterstaining, the cells were then incubated for 1 h with a secondary antibody solution (1:500 Goat anti-mouse Alexa 647, #A28181, ThermoFisher) containing Phalloidin i488 (1:1000, ab176753, Abcam) and DAPI (1:1000 from 10 mM stock). Stacked images were taken on a Leica Mica system, and images were analysed using LasX software (Leica). The experiments were done in triplicates and repeated at least two times.

### 2.8 Statistical analysis

The half maximal inhibitory concentrations (IC_50_) for *G. duodenalis* trophozoites and the 50% cytotoxic concentrations (CC_50_) were determined using non-linear regression analysis; the selectivity index (SI) was calculated as the ratio of IC_50_ to CC_50_ for each *G. duodenalis* isolate in relation to both MDCK and Caco-2 cell lines. Drug cytotoxicity against mammalian cells was assessed by calculating the percentage of viable cells, normalizing the absorbance of treated wells to the vehicle control set at 100% viability. Results were presented as mean ± standard deviation (SD) and analysed using one or two-way ANOVA with Tukey's multiple comparison test. A p-value of less than 0.05 was considered statistically significant. All the analyses were performed using GraphPad Prism® version 9 (GraphPad Software, San Diego, USA).

## 3 Results

### 3.1 Activity of T. avellanedae extracts and β-lapachone against G. duodenalis trophozoites, Caco-2 and MDCK cell lines

*T. avellanedae* dry (TD) and hydroalcoholic (TH) extracts both displayed effective activity against *G. duodenalis* trophozoites, with comparable 48-h IC_50_ values irrespective of the isolate or the Assemblage tested (Table 2). Additionally, β-lapachone exhibited potent antigiardial activity, in agreement with a previous report (Corrêa et al., 2009). Both β-lapachone and MTZ showed a broader range of IC_50_ values across the isolates tested, particularly concerning the laboratories where they were maintained (Table 2). Noteworthy, β-lapachone proved to be more potent than MTZ, consistently displaying slightly lower 48-h IC_50_ values per isolate, with tight confidence intervals for all measurements suggesting high data reliability.

**Table 2:** Activity of *Tabebuia avellanedae* dry extract (TD), *Tabebuia avellanedae* hydroalcoholic extract (TH), β-lapachone and metronidazole (MTZ) against *Giardia duodenalis* trophozoites.

	WBC6∗	WBC6°	GS/M∗	GS/M°	P424/A5°
**TD** (mg/ml)					
IC_50_ (95% CI)	1.50 (1.45–1.55)	1.32 (1.21–1.43)	1.28 (1.24–1.33)	1.16 (1.12–1.19)	1.08 (1.06–1.10)
**TH** (mg/ml)					
IC_50_ (95% CI)	1.38 (1.34–1.42)	1.27 (1.15–1.40)	1.19 (1.16–1.22)	1.19 (1.14–1.24)	1.14 (1.11–1.17)
**β-lapachone** (μM)					
IC_50_ (95% CI)	6.2 (4.41–7.04)	2.43 (2.18–2.71)	5.90 (5.26–6.71)	3.97 (3.51–4.42)	3.16 (2.81–3.56)
**MTZ** (μM)					
IC_50_ (95% CI)	6.77 (6.11–7.50)	4.67 (4.07–5.35)	7.77 (7.23–8.36)	6.55 (5.36–8.00)	5.13 (4.36–6.03)

a) Antigiardial activity is expressed as inhibitory concentration 50 (IC_50_) with a 95% Confidence Interval (CI) for each compound. IC_50_ values are expressed in mg/ml for TD and TH and in μM for β-lapachone and MTZ. Each experiment was done in triplicate, and at least three biological replicates were performed. ∗ = *G. duodenalis* isolates maintained in the IT laboratory; ° = *G. duodenalis* isolates maintained in the DE laboratory.

The compounds were then tested against mammalian epithelial cell lines of human and canine origin to evaluate their potential cytotoxicity. Caco-2 and MDCK cell lines were chosen as both have been widely used to assess the permeability of compounds in *vitro* conditions for pharmaceutical use (Jin et al., 2014; Kus et al., 2023), and because Caco-2 cells are commonly used for *in vitro* interaction studies with *G. duodenalis* trophozoites (Kraft et al., 2017). TD showed cytotoxic effects for both cell lines at concentration higher than 1 and 2 mg/ml, respectively, with a limited effect with respect to the incubation time (Supplementary Figs. S1 and S2). The 48-h CC_50_ for both cell lines (Supplementary Fig. S3) was in the same range as 48-h IC_50_ measured for *G. duodenalis* trophozoites (Table 2, Table 3). β-lapachone also exhibited strong cytotoxic effects in both immortalised cell lines within the low μM range (Supplementary Figs. S1 and S2). Similar to TD, the 48-h CC_50_ for β-lapachone was of the same order of magnitude as the 48-h IC_50_ for *G. duodenalis.* Based on these results, a mean SI < 1.7 was obtained for TD and β-lapachone for either of the cell lines (Table 4). Thus, under these assumptions, the compounds would be judged as unsuitable as antigiardial agents. In contrast, TH did not demonstrate significant cytotoxicity in either cell line, with SI for TH > 100 since no CC_50_ could be calculated (Table 4). Although a trend toward reduced cell viability was noted in MDCK cells at the highest concentration, however, this was not statistically significant (Supplementary Figs. S1 and S2).

**Table 3:** Activity of *Tabebuia avellanedae* dry extract (TD), *Tabebuia avellanedae* hydroalcoholic extract (TH), and β-lapachone against Caco-2 and MDCK cell lines.

	Caco-2	MDCK
**TD** (mg/ml)		
CC_50_ (95% CI)	1.71 (1.51–1.92)	1.73 (1.56–1.93)
**TH** (mg/ml)		
CC_50_ (95% CI)	>100	>100
**β-lapachone** (μM)		
CC_50_ (95% CI)	4.19 (3.41–5.14)	4.11 (3.69–4.58)

a) Cytotoxicity was measured after 48 h and expressed as cytotoxicity concentration 50 (CC_50_) with a 95% Confidence Interval (CI) for each compound. CC_50_ values are expressed in mg/ml for TD and TH and in μM for β-lapachone. Each experiment was done in triplicate, and at least three biological replicates were performed.

**Table 4:** Selectivity index (SI) of each compound for each *G. duodenalis* isolate in relation to MDCK and Caco-2 cell lines.

	WBC6^a^	WBC6°	GS/M^a^	GS/M°	P424/A5°
TD					
MDCK	1.16	1.32	1.35	1.50	1.61
Caco-2	1.14	1.30	1.33	1.47	1.58
**TH**					
MDCK	>100	>100	>100	>100	>100
Caco-2	>100	>100	>100	>100	>100
**β-lapachone**					
MDCK	0.66	1.69	0.70	1.04	1.30
Caco-2	0.68	1.72	0.71	1.06	1.32

a = *G. duodenalis* isolates maintained in the IT laboratory; ° = *G. duodenalis* isolates maintained in the DE laboratory.

### 3.2 UHPLC-IMS-HRMS analysis of TH identifies potential antigiardial molecules

Following the promising results obtained with TH, the profile of metabolites present in the extract was screened by untargeted metabolomic fingerprinting approach using the combination of Ultra-Performance Liquid Chromatography-Ion Mobility Separation coupled to High Resolution Mass Spectrometry (UHPLC-IMS-HRMS*)*. Supplementary Fig. S4 shows the Base Peak Intensity (BPI) chromatogram of the TH*.* After applying the “Binary Sample Compare” tool, a list of 45 candidate mass metabolites was found in TH by the UNIFI software. After querying the “Natural Products Profiling CCS” online library, a shortlist of 22 metabolites was identified with high confidence through the combination of ion mobility experiments, accurate mass determination, and mass spectra, even without the comparison of retention times, fragmentations, and CCS values with commercially available standards (Table 5). Noteworthy, β-lapachone was among the identified metabolites, but the low toxicity of TH in mammalian cells compared to purified β-lapachone might suggest that the presence of other metabolites either partially counteract the cytotoxic effect of β-lapachone in mammalian cell or indicates that β-lapachone is present at sub-toxic concentration for mammalian cells, with its efficacy against *G. duodenalis* improved by other metabolites.

**Table 5:** List of metabolites identified through untargeted screening of *Tabebuia avellanedae* hydroalcoholic extract (TH)^a^.

No.	Component name	Theoretical neutral mass (Da)	Experimental neutral Mass (Da)	Mass error (mDa)	m/z	RT (min)	Theoretical fragments found	Adducts	Theoretical CCS (Å^2^)	Experimental CCS (Å^2^)	CCS Delta Error (%)
1	(−)-Ouabain	584.2833	584.285	1.3	585.292	6.84	2	+H	236.48	237.76	0.54
2	(±)-Abscisic acid	264.1362	264.136	0.2	265.144	3.81	1	+H	156.12	158.74	1.68
3	Auraptene	298.1569	298.154	−3	299.161	11.74	1	+H	168	169.44	0.86
4	Austricin	262.1205	262.12	−1	263.127	4.68	2	+H	156.72	156.33	−0.25
5	Carnitine	161.1052	161.105	0	162.112	0.57	2	+H	132.59	134.29	1.28
6	Dehydrocostus lactone	230.1307	230.131	−0.2	231.138	5.26	2	+H	150.67	153.04	1.57
7	Eriodictyol	288.0634	288.064	0.6	289.071	2.82	1	+H	164.33	167.31	1.81
8	Flavokawain A	314.1154	314.114	−1.9	315.121	2.49	4	+H	172.7	173.93	0.71
9	Flavokawain B	284.1049	284.106	0.7	285.113	2.64	1	+H	163.94	164.39	0.28
10	Hydrocotarnine	221.1052	221.105	0	222.113	0.6	4	+H	146.99	147.41	0.29
11	Indirubin	262.0742	262.077	2.3	263.084	4.9	1	+H	151.7	152.16	0.3
12	Isorhamnetine-3-glucoside	478.1111	478.115	3.8	479.122	2.5	1	+H	207.49	207.34	−0.07
13	Isorhamnetine-3-rutinoside	624.169	624.173	4	625.18	3.84	1	+H	234.77	237.76	1.27
14	Luteolin	286.0477	286.048	0.1	287.055	2.19	1	+H	159.75	158.68	−0.67
15	Mycophenolic acid	320.126	320.124	−2.5	321.131	2.51	2	+H	166.33	163.26	−1.84
16	Phloretin	274.0841	274.084	−0.6	275.091	5	1	+H	159.76	158.98	−0.49
17	Robinetine	302.0427	302.041	−1.8	303.048	0.61	1	+H	164.51	163.12	−0.85
18	Salsoline	193.1103	193.111	0.3	194.118	0.6	1	+H	144.86	143.08	−1.23
19	Scopoletin	192.0423	192.042	−0.4	193.049	2.48	1	+H	133.33	134.26	0.7
20	Sinensetine	372.1209	372.118	−2.6	373.126	2.47	1	+H	187.17	189.47	1.23
21	Stachydrine	143.0946	143.095	0.4	144.102	0.58	1	+H	127.83	125.69	−1.67
22	β-Lapachone	242.0943	242.095	0.3	243.102	11.72	1	+H	150	148.36	−1.09

a)Analysis was performed twice.

### 3.3 Activity of T. avellanedae extracts and β-lapachone on non-infected ODM and ODM infected with G. duodenalis trophozoites

As organoid derived primary cells represent a more physiological model than immortalised cell lines, we used this model for further evaluating the cytotoxicity of the compounds and to determine their anti-*G. duodenalis* efficacy under co-culture conditions with host cells. Our well-characterized ODM model is based on a trans-well filter that generates strongly polarised and very tight epithelial layers akin to intestinal epithelia (Holthaus et al., 2021, 2022; Weiss et al., 2022). In the framework of this study the model has three major advantages: i) β-lapachone is a known anti-cancer drug, and thus its effects on cancer-derived cell lines like Caco-2 cannot be correlated with potential effects on primary cells; ii) ODM consist of differentiated primary intestinal epithelial cells that are resistant to TYI-S-33, making them suitable for conducting infection experiments under optimal conditions for *G. duodenalis* without harming the host cells (Holthaus et al., 2022); iii) the cells in the ODM system are strictly polarised with proper junction complexes, thus more accurately mimicking the epithelial barrier (Weiss et al., 2022).

**Fig. 1:**
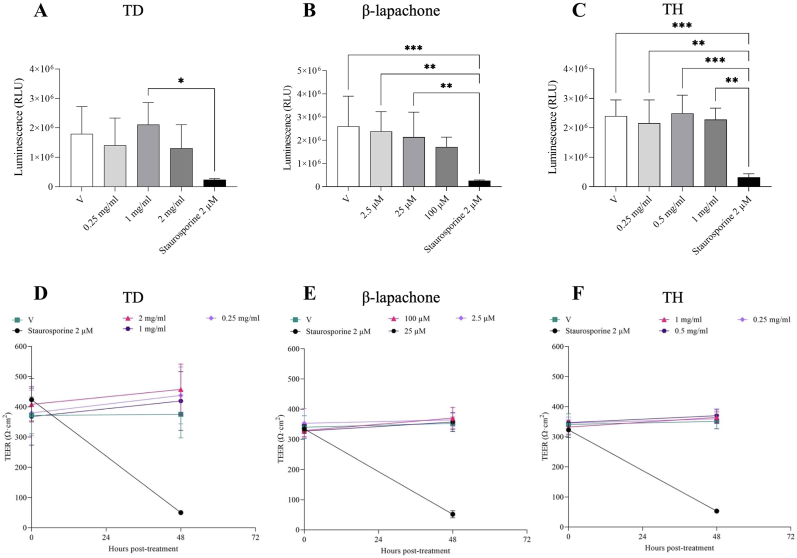
**Effects of different concentrations of *Tabebuia avellanedae* dry extract (TD), hydroalcoholic extract (TH) and β-lapachone on ODM.** A-C) ODM viability. Staurosporine at 2 μM, a nonselective apoptosis inducer, was used as positive control in all respective experiments (Holthaus et al., 2022), along with vehicle (DMSO and EtOH) control (V). After 48 h, cell viability was assessed using a luminescent assay to measure ATP levels in the culture as an indicator of metabolically active cells. Each experiment was done in duplicate and repeated at least twice. Results are expressed as mean ± SD and were analysed by one-way analysis of variance (ANOVA) followed by Tukey's multiple comparison test. Statistical significance was set at p < 0.05; ∗∗∗p < 0.001, ∗∗p < 0.01, ∗p < 0.05 vs vehicle control (V), as indicated in figure. D–F) Transepithelial electric resistance (TEER) of ODM following 48 h of treatment with vehicle (V) or the indicated compounds: *T. avellanedae* dry extract (TD), *T. avellanedae* hydroalcoholic extract (TH) and β-lapachone. Blank electric resistance (cell-free trans-well insert) was subtracted from raw resistance values and standardised for 1 cm^2^ surface area. Each experiment was done in duplicate and repeated at least twice with similar results.

**Fig. 2:**
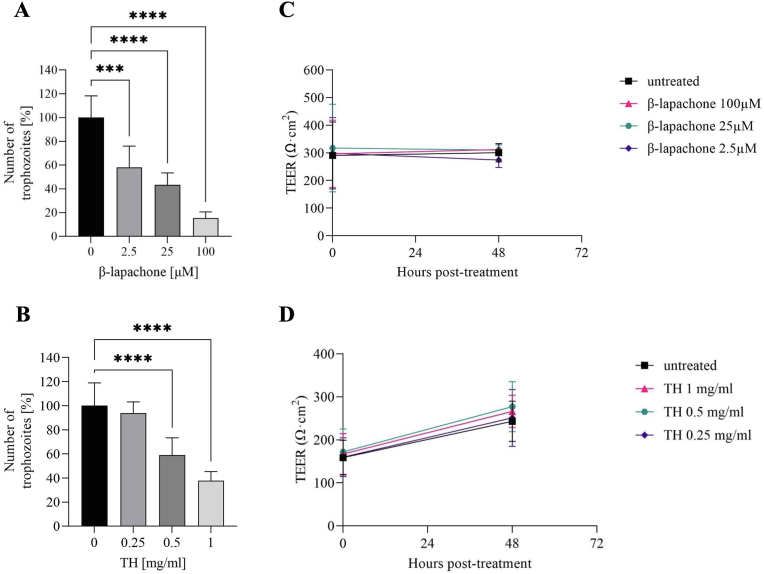
**Evaluation of β-lapachone and *Tabebuia avellanedae* hydroalcoholic extract (TH) on *Giardia duodenalis* WBC6 trophozoites cultured on ODM.** A-B) Parasite count after 48 h of culture with or without the indicated compounds at different concentrations. Statistical significance was set at p < 0.05; ∗∗∗∗p < 0.0001, ∗∗∗p < 0.001, ∗∗p < 0.01, ∗p < 0.05 vs 0 μM or 0 mg/ml, as indicated in figure. Each experiment was done in duplicate and repeated at least twice. C-D) Transepithelial electric resistance (TEER) evaluation in ODM after 48 h of culture with *G. duodenalis* trophozoites, with or without treatment using the indicated compounds at different concentrations. Blank electric resistance (cell-free trans-well insert) was subtracted from raw resistance values and standardised for 1 cm^2^ surface area. Representative experiments conducted in triplicates are shown. Each experiment was done in duplicate and repeated at least twice with similar results.

**Fig. 3:**
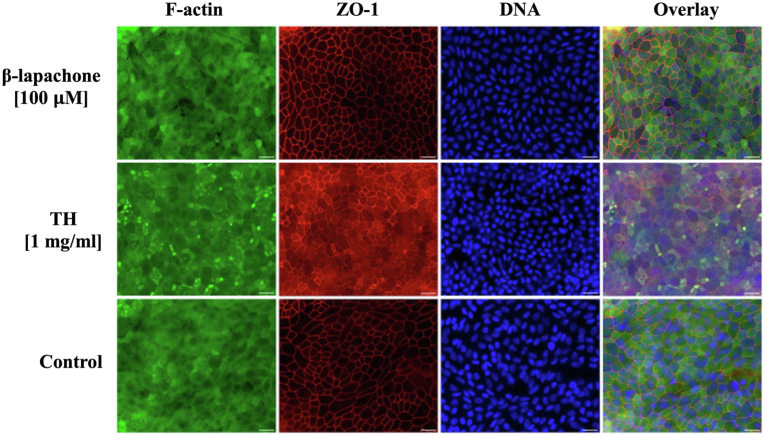
**Immunofluorescence analysis of ODM 48 h post *G. duodenalis* infection and various treatment regimens shows similarly intact barrier integrity.** ODM were infected with 2x10^5^ trophozoites (WBC6) and treated with β-lapachone or *T. avellanedae* hydroalcoholic extract (TH) as indicated, or non-treated (control) for 48 h. After detachment of the parasites, the filters were fixed and stained with Phalloidin (F-actin, green), anti-ZO-1 antibody (ZO-1, red) and DAPI (DNA, blue). Images were captured on a Leica Mica imaging system with optimised optical stacks and processed using Leica Las X software. Note that, due to the polarised nature of the ODM, nuclei are presented from separate Z-layers. Further, the filters are derived from different experiments and staining procedures, resulting in varying image quality. However, the images highlight comparable and intact barrier integrity across all samples. Each experiment was done in duplicate and repeated at least twice with similar results.

Compounds at any of the three chosen concentrations did not affect the metabolic activity of ODM cells, suggesting, in contrast to the immortalised cell lines, no cytotoxicity towards the ODM in comparison to the vehicle control (Fig. 1). Furthermore, the monolayer barrier integrity was monitored using TEER measurements, which showed no impairment upon treatment with the tested compound concentrations (Fig. 1). Since TH proved to be a safer and more standardised formulation compared to TD, we next tested the effect of β-lapachone and TH on ODM infected with *G. duodenalis* trophozoites to determine whether the drug response would be comparable to the result without host cells. We used a trophozoite concentration known not to affect host cell barrier integrity in the tested time frame (Holthaus et al., 2022). The results indicate that both β-lapachone and TH (Fig. 2) effectively reduce the number of trophozoites as their concentrations increase, without affecting host cell barrier integrity as measured by TEER and immunofluorescence microscopy after 48 h of treatment (Fig. 2, Fig. 3).

## 4 Discussion

Despite the global prevalence and impact of *G. duodenalis* on public health, effective drug treatments for giardiasis are limited and associated with treatment failures. Here, we provide evidence that compounds from the known medicinal plant *T. avellanedae* possess effective antigiardial activity *in vitro*, which may offer potential new treatment options. Both plant extracts and the active compound β-lapachone, also present in the hydroalcoholic extract of *T. avellanedae*, as confirmed by our metabolites analysis, were effective against isolates from the two zoonotic *G. duodenalis* Assemblages AI (WBC6) and B (GS/M and P424/A5). *T. avellanedae* hydroalcoholic extract and β-lapachone proved to be safe when tested on ODM, which mimics physiological conditions more closely than commonly used epithelial cell lines (i.e., Caco-2 and MDCK). Moreover, for the first time, the efficacy of new potentially antigiardial compounds was confirmed in a *Giardia*-organoid co-culture system.

Concerning the efficacy of the different compounds on *G. duodenalis*, the IC_50_ values measured for TD and TH are in mg/ml range, which appears to be a relatively high concentration when compared to other reports on natural hydroalcoholic extracts tested against *G. duodenalis* (Kabbashi et al., 2015; Pintong et al., 2020). However, the limited number of studies on the efficacy of natural products against *G. duodenalis* do not allow any strong conclusions. Most of the research has focused on essential oils or aqueous extracts, with compounds primarily tested on cysts rather than trophozoites (Fallahi et al., 2016; Harba et al., 2019). The relatively similar IC_50_ values of both extracts on *G. duodenalis* trophozoites suggest no differences in solubility of active metabolite(s) in the two solvents used (DMSO or ethanol), although β-lapachone has a higher solubility in alcoholic solvents compared to water-based solvents (Kim et al., 2018). This also suggests that a rather complex mixture of active metabolites in both extracts could mediate the reported antigiardial effect in addition to β-lapachone. Our metabolite analysis of TH supports, with high confidence, the occurrence of 22 metabolites, confirming the presence of β-lapachone as well as luteolin, a flavonoid found in another extract tested against *G. duodenalis* that has shown effectiveness (Palomo-Ligas et al., 2022). Lutein's mechanism of action appears to cause damage to the cytoskeleton due to alterations in the expression and distribution of α-tubulin, particularly in the ventral disk, which is a key structure for adhesion and pathogenesis (Palomo-Ligas et al., 2022). Corrêa et al. (2009) found that treatment with β-lapachone in *G. duodenalis* induces several apoptotic morphological changes, such as cell shrinkage, chromatin condensation, membrane blebbing, and vacuolization. Although the parasite is amitochondrial, β-lapachone exhibits characteristics of both apoptotic and autophagic cell death, suggesting a complex mechanism of action in *Giardia* (Corrêa et al., 2009). In cancer cells, β-lapachone has been shown to alter the cell redox state by undergoing a futile NAD(P)H:quinone oxidoreductase 1 (NQO1)-mediated redox cycle, which results in high levels of superoxide and subsequent peroxide formation that eventually kill the cell (Pink et al., 2000). A similar mechanism of action for β-lapachone can also be hypothesized for G*. duodenalis*. Indeed, another naphthoquinone, 2-methy-1,4-naphthoquinone (menadione) is highly toxic for *G. duodenalis* under microaerophilic condition by redox cycling and ROS generation (Paget et al., 2004). Although the antigiardial activity of other redox cycler compounds, such as NBDHEX, has been also demonstrated (Lalle et al., 2015), the exact mechanism of action of β-lapachone has yet to be proven. Furthermore, among the 22 identified metabolites, some have been effectively tested against other parasites, such as auraptene against *Haemonchus contortus* (Dugan et al., 2024), eriodictyol (specifically 6,8-diprenyleriodictyol) against intracellular amastigotes of *Leishmania amazon*e*nsis* (Sandjo et al., 2016), flavokawain B against *Trypanosoma cruzi* and *Trypanosoma brucei*, and non-toxic to Hep G2 cells (Rodrigues et al., 2017), as well as indirubin (Efstathiou et al., 2018). It would be interesting to test the efficacy of these molecules also against *G. duodenalis.*

For the first time, the efficacy of β-lapachone was compared to the reference drug MTZ, the first choice for the treatment of giardiasis (Gardner and Hill, 2001; Leung et al., 2019), showing a slightly lower IC_50_ value for β-lapachone than for MTZ in all *G. duodenalis* isolates tested. We tested various isolates, as previous studies have noted significant variability in drug susceptibility between assemblages (Bénéré et al., 2011), and we confirmed differences in the determined IC_50_ values between isolate WBC6 (Assemblage AI) and the Assemblage B isolates (GS/M, P424/A5). Notably, we tested all components with WBC6 and GS/M isolates maintained in two laboratories and encountered only minimal differences in the results, highlighting the robustness of our findings. The observed variability in IC_50_ for both MTZ and β-lapachone between Assemblages and laboratory isolates batch is consistent with data reported for MTZ in WBC6 by different authors, with 48-h IC_50_ ranging from 2.1 to 8.5 μM (as measured by ATP content assay) (Valdez et al., 2009; Chen et al., 2011; Tejman-Yarden et al., 2011; Debnath et al., 2014).

Assessment of drug safety on an appropriate *in vitro* cellular model is a fundamental step in the process of selecting the most promising drug candidates to progress to pre-clinical *in vivo* test. Our results suggest that Caco-2 and MDCK epithelial cell lines might not be appropriate, despite their common use for the pharmaceutical evaluation of drug permeability (Volpe, 2011). While TH did not exhibit significant cytotoxic effects in either cell line at any time point, with a SI > 100, TD and, in particular, β-lapachone were shown to be cytotoxic. The cytotoxic effect of β-lapachone has indeed been observed in various human carcinoma cell lines, such as oral squamous cell carcinoma, hepatocellular carcinoma, and gastric and colon adenocarcinoma (Gomes et al., 2021; Zhao et al., 2024). Similar to findings in several epithelial cell lines (Kung et al., 2008), no prior evidence of β-lapachone cytotoxicity has been reported for MDCK (Nomura et al., 2014). However, different studies have also shown inconsistent results across various cell lines (Pereira et al., 2006; Lima Bezerra et al., 2022). Few studies have reported on testing of *T. avellanedae* alcoholic extracts both *in vitro* and *in vivo*, even if with contradictory results. While *T. avellanedae* ethanol extract showed no cytotoxicity in macrophage-like (RAW264.7) and chondrosarcoma (SW1353) cell lines (Park et al., 2017), a methanol extract was toxic to human tumor cell lines but not to healthy cells (Pires et al., 2015). Lemos et al. (2012) reported increased DNA damage in liver cell nuclei of rats treated with a hydroalcoholic extract compared to controls. In contrast, de Miranda et al. (2001) found that an aqueous extract of *T. avellanedae* inner bark was not acutely toxic in mice at doses up to 5000 mg/kg. The discordant observation of these studies regarding toxicity can be linked to variation in incubation time with the compound, concentration or dosage applied, variation in metabolite composition due to plant species and geographical location, or extraction methods (Lima Bezerra et al., 2022).

The use of organoids is becoming a new standard for drug screening, as they can replicate more physiological conditions or even allow for personalized medicine when derived from patient (Wang and Jeon, 2022). Here, we confirmed that a well-established and characterized human duodenal ODM infection model (Holthaus et al., 2021, 2022; Weiss et al., 2022) provides robust evidence of the safety of all the compounds at any of the tested concentrations, with no significant cytotoxicity over a 48-h period and without disrupting intestinal epithelial cell integrity, as indicated by no significant reduction of TEER measurements. In the view of better compliance with the 3 R approach, we have also shown that human duodenal ODM infected with *G. duodenalis* can simultaneously provide valuable information on both drug efficacy against the parasite and safety for host cells, minimizing confounding effects of infection on drug effect. Indeed, the ODM were infected with a MOI of 1 (2x10^5^), as this MOI did not show any barrier disruption or TEER decrease in a previous study (Holthaus et al., 2022). Both β-lapachone and TH have demonstrated effective reductions in *G. duodenalis* trophozoites, showing dose-dependent activity without any reduction in TEER or barrier destruction observed in immunofluorescence analysis. In comparison to tests performed in axenic *in vitro* conditions (Table 2), the IC_50_ values for WBC6 trophozoites treated with both β-lapachone (7.2 μM; 95% CI 3.7 to 14.4) and TH (1.4 mg/ml; 95% CI 1.2 to 1.6) (Supplementary Fig. S5) were slightly higher, which may be due to assay-related differences (e.g., medium volume, apical treatment selection in the trans-well assay) or possible metabolization of the compounds by host cells. Furthermore, the higher IC_50_ values observed in the ODM model suggest an increased tolerance of trophozoites in the presence of host cells compared to axenic culture. This finding aligns with previous research suggesting that *G. duodenalis*, and other intestinal parasites, exhibits enhanced viability when interacting with host cells (Costa et al., 2000; Nash, 2019). This protective effect is likely due to the host-parasite interactions, which may provide a more supportive environment for parasite survival (Nash, 2019).

## 5 Conclusions

In conclusion, our *in vitro* results highlight the therapeutic potential of *T. avellanedae* against *G. duodenalis*. This natural extract and β-lapachone warrant further investigation as a novel antigiardial therapeutic; an in-depth exploration of their mechanism of action could unveil valuable therapeutic strategies that would contribute to the fight against drug failures and help prevent drug resistance. Additionally, this study marks the innovative utilisation of stem cell based ODM technology for evaluating antigiardial treatments, as it allows co-culturing and assessment under more physiological conditions than previous model using immortalised cell lines.

## CRediT authorship contribution statement

**Giulia Rigamonti:** Writing – review & editing, Writing – original draft, Visualization, Methodology, Investigation, Formal analysis, Data curation. **Fabrizia Veronesi:** Writing – review & editing, Writing – original draft, Visualization, Methodology, Investigation, Funding acquisition. **Elisabetta Chiaradia:** Writing – review & editing, Writing – original draft, Visualization, Methodology, Investigation. **Petra Gosten-Heinrich:** Resources, Investigation, Formal analysis. **Antonia Müller:** Methodology, Investigation, Formal analysis. **Leonardo Brustenga:** Writing – original draft. **Stefano de Angelis:** Resources. **Alessia Tognoloni:** Writing – review & editing, Writing – original draft, Investigation. **Riccardo De Santo:** Writing – review & editing, Writing – original draft, Methodology, Investigation. **Christian Klotz:** Writing – review & editing, Writing – original draft, Visualization, Supervision, Formal analysis, Conceptualization. **Marco Lalle:** Writing – review & editing, Writing – original draft, Visualization, Validation, Supervision, Resources, Methodology, Funding acquisition, Conceptualization.

## Funding

This work was co-funded by the European Union’s Horizon Europe Project 101136346 EUPAHW

## Declaration of competing interest

Giulia Rigamonti, Fabrizia Veronesi, Elisabetta Chiaradia, Petra Gosten-Heinrich, Antonia Müller, Leonardo Brustenga, Alessia Tognoloni, Riccardo De Santo, Christian Klotz and Marco Lalle declare no competing interest concerning the research, authorship, publication of this article and/or financial and personal relationships that could inappropriately influence this work.

Stefano de Angelis is manager at Deakos SRL who provided access to the test compounds *Tabebuia avellanedae* TD and TH.
