# Supplementary material for: Selective activity of Tabebuia avellanedae against Giardia duodenalis infecting organoid-derived human gastrointestinal epithelia
Source: Int J Parasitol Drugs Drug Resist. 2025 Jan 22;27:100583. doi: 10.1016/j.ijpddr.2025.100583 (PMC11802375; doi:10.1016/j.ijpddr.2025.100583)
Supplement: Multimedia component 1 [file mmc1.pdf]

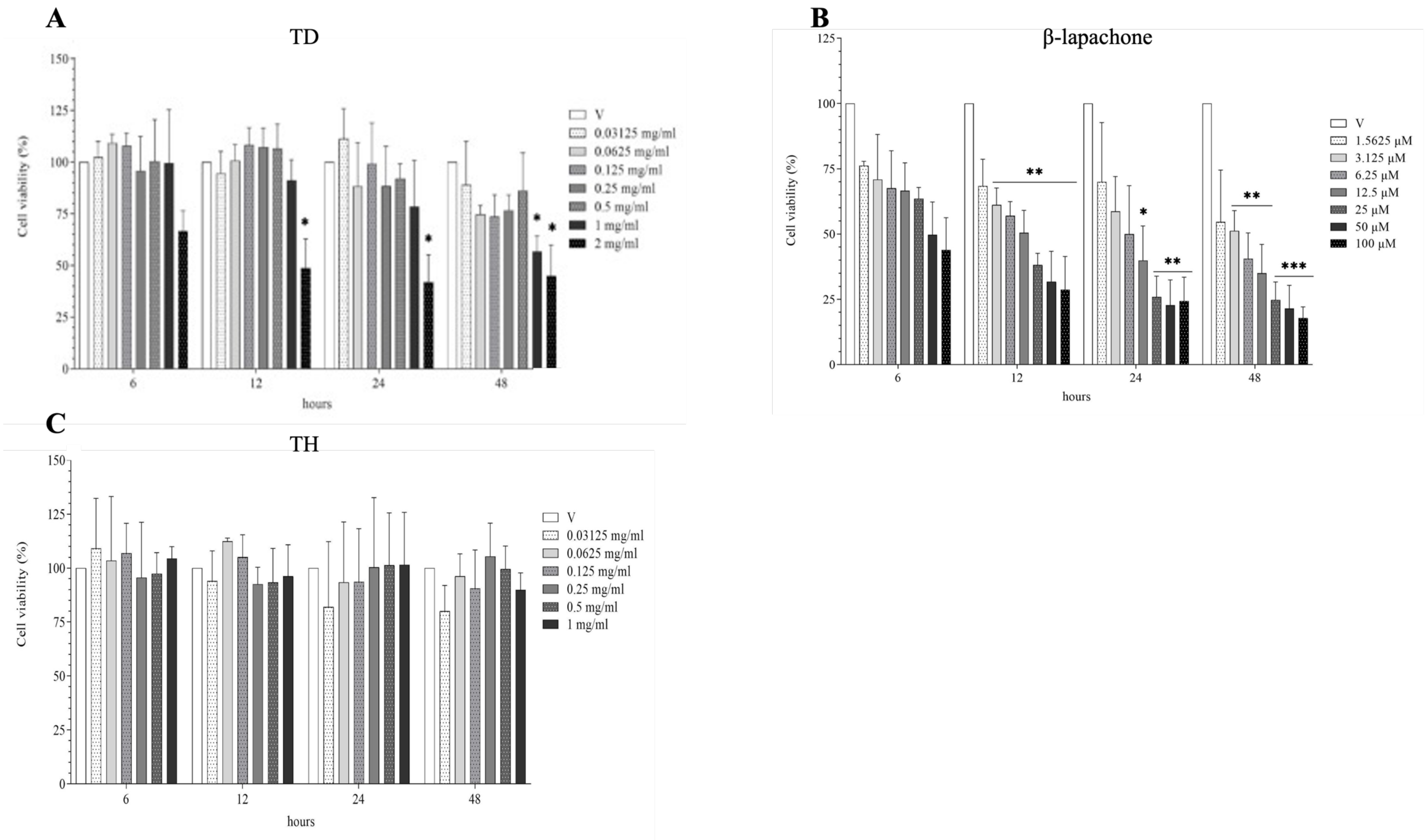

Supplementary Figure S1. Effects of different concentrations of *Tabebuia avellanedae* dry extract (TD) (A), β-lapachone (B), and *Tabebuia avellanedae* hydroalcoholic extract (TH) (C) on Caco-2 cell viability after 6, 12, 24, and 48 hours, tested using the CCK8 assay. The percentage of cell viability was calculated as the ratio between the optical density (OD) of the cells treated with specific compounds and the OD of the control cells. ***$p<0.001$,

**p<0.01, *p<0.05 vs vehicle control (V), as indicated in figure. Each experiment was done in triplicate, and at least three biological replicates were performed.

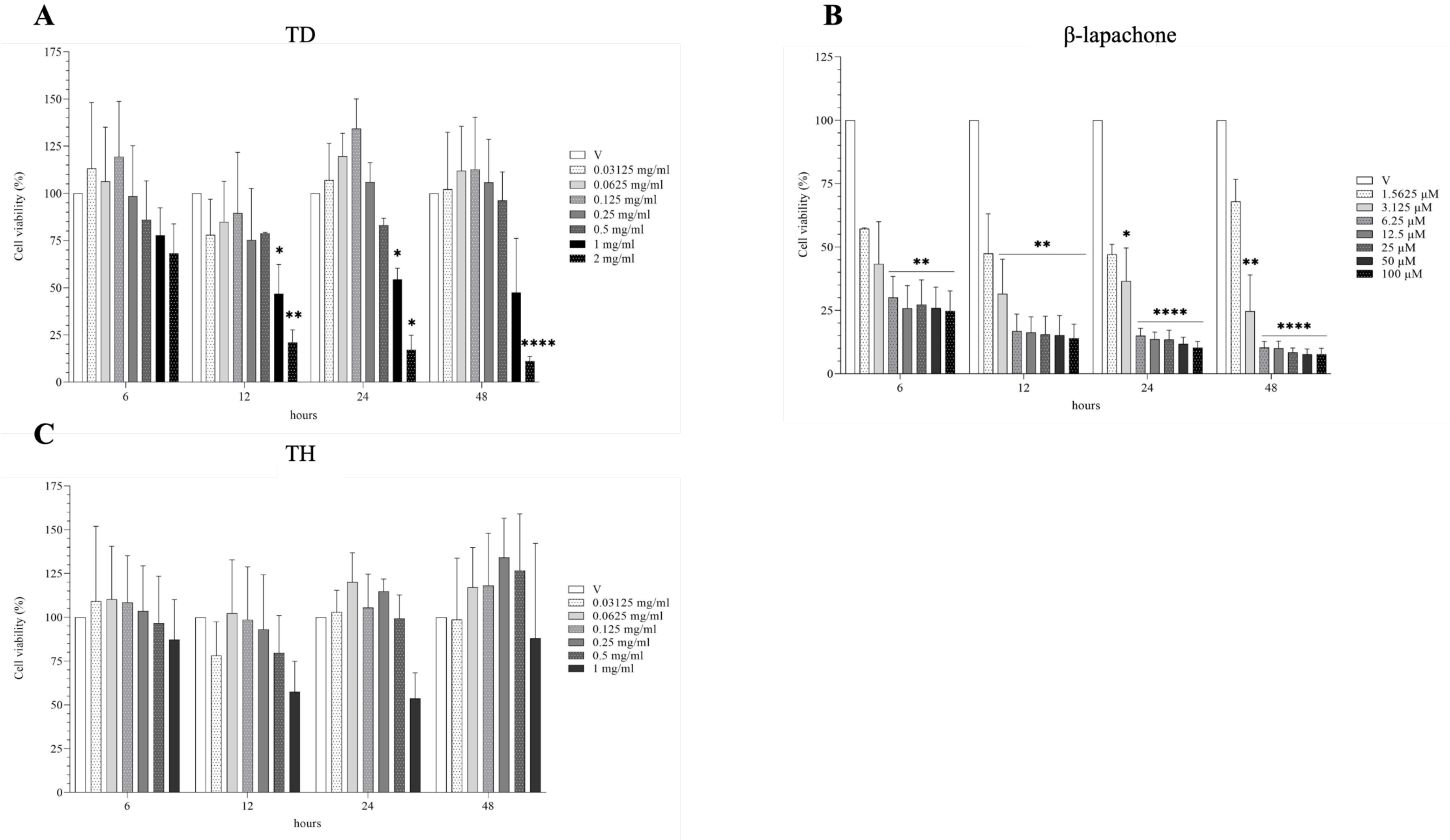

Supplementary Figure S2. Effects of different concentrations of *Tabebuia avellanedae* dry extract (TD) (A), β-lapachone (B), and *Tabebuia avellanedae*

hydroalcoholic extract (TH) (C) on MDCK cell viability after 6, 12, 24, and 48 hours, tested using the CCK8 assay. The percentage of cell viability was calculated as the ratio between the optical density (OD) of the cells treated with specific compounds and the OD of the control cells. ***$p<0.001$, **$p<0.01$, *$p<0.05$ vs vehicle control (V), as indicated in figure. Each experiment was done in triplicate, and at least three biological replicates were performed.

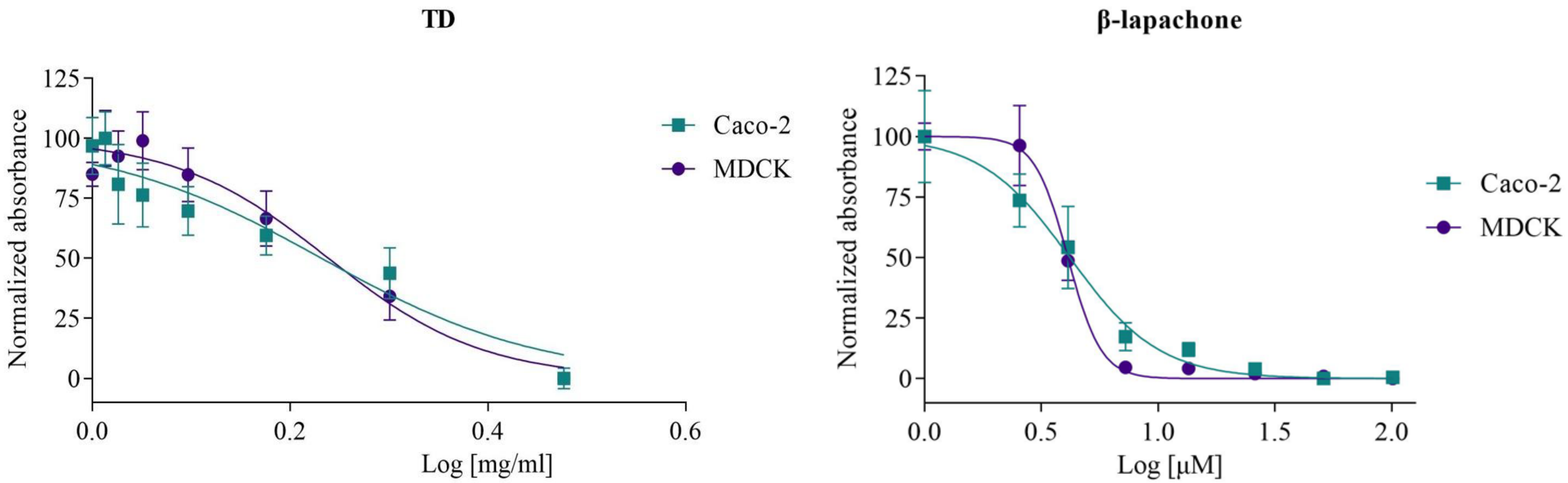

Supplementary Figure S3. The 50% cytotoxic concentrations (CC_{50}) curves of *Tabebuia avellanedae* dry extract (TD) and β-lapachone against MDCK and Caco-2 cell lines at 48 hours. The CC_{50} values were determined using non-linear regression analysis in GraphPad Prism® version 9 (GraphPad Software, San Diego, USA). Each experiment was done in triplicate, and at least three biological replicates were performed.

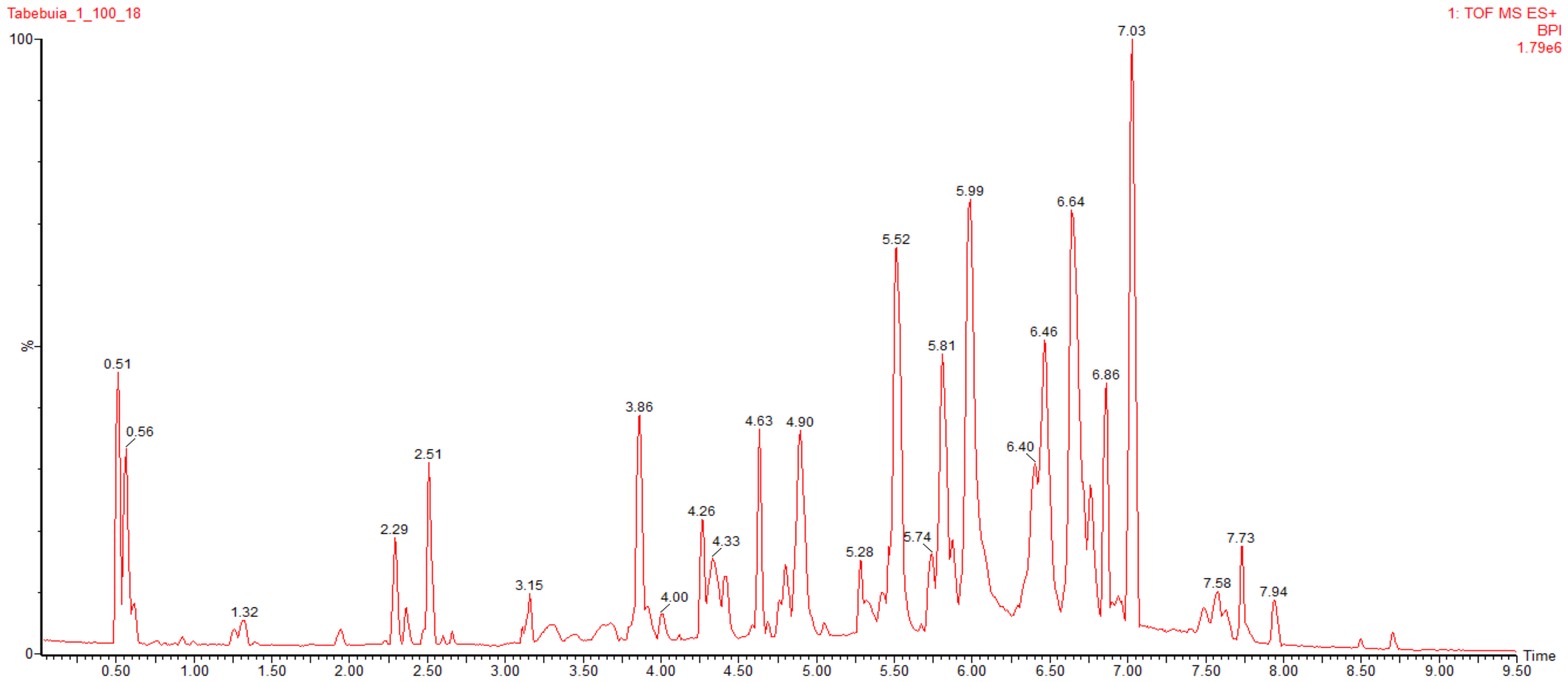

Supplementary Figure S4. Base Peak Intensity (BPI) chromatogram of *Tabebuia avellanedae* hydroalcoholic extract (TH). Analysis was performed twice

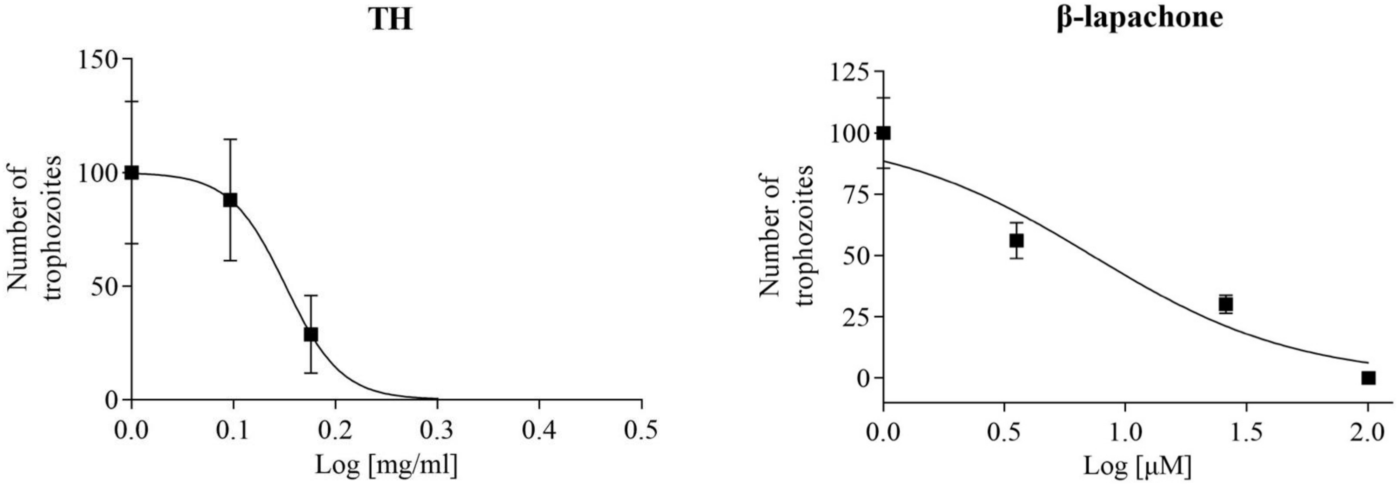

Supplementary Figure S5. The half maximal inhibitory concentrations (IC_{50}) curves of *Tabebuia avellanedae* hydroalcoholic extract (TH) and β-lapachone against *G. duodenalis* trophozoites cultivated on ODMs for 48 hours. The IC_{50} values were determined using non-linear regression analysis in GraphPad Prism® version 9 (GraphPad Software, San Diego, USA). Each experiment was done in duplicate and repeated at least twice with similar results.
